# Complex barite filter cake removal using in-situ generated acids by thermochemicals

**DOI:** 10.1038/s41598-020-72858-y

**Published:** 2020-09-25

**Authors:** Badr S. Bageri, Ibrahim Gomaa, Mohamed Mahmoud, Shirish Patil, Ayman Al-Nakhli

**Affiliations:** 1 Department of Petroleum Engineering, King Fahd University of Petroleum & Minerals, Dhahran, Saudi Arabia; 2 EXPEC ARC, Production Technology Division Saudi Aramco, Dhahran, Saudi Arabia

**Keywords:** Analytical chemistry, Catalysis, Energy, Polymer chemistry

## Abstract

In sandstone formations, the quartz particles integrate with drilling fluid solids and become part of the filter cake structure. As a result, the dissolution rate of the filter cake diminishes and reduces the removal efficiency. This paper presents a novel solution to overcome the challenges that restricts the filter cake removal process such as the presence of the quartz layer and the polymer coat. A multi-stage method for removing the filter cake from a wellbore is presented. The composition of the new formulation is; ammonium fluoride (NH_4_F), with a strong oxidizer, such as sodium bromate (NaBrO_3_) causes an exothermic reaction in the first stage, thereby removing the quartz layer and polymer coat in the filter cake by the in-situ generated HF acid. During the second stage for the barite-based filter cake, chelating agents combined with convertor catalysts were used to dissolve the barite. Solubility experiments were conducted to evaluate the efficiency at each stage in the filter cake removal process at 300 ºF and 500 psi. The experimental results showed that the formulation consisting of ammonium fluoride (NH_4_F), with a strong oxidizer (sodium bromate,NaBrO_3_), combined with exothermic reaction was able to generate HF in-situ, which in turn dissolved the quartz mineral and remove the polymer from the filter cake.

## Introduction

During overbalance drilling operations, drilling fluid is used for one or more specific functions such as cooling and lubricating the drilling, carrying the cuttings away from the bit, and stabilizing and supporting the heaving formations (shales) and preventing them from flowing back into the well. In addition to these functions, the drilling fluid should be designed to impose a sufficiently high hydrostatic pressure, and hinder any fluids or solids from entering the formation (lost circulation), which may prevent hydrocarbon flow towards the well^1–6^. In order to achieve these functions, the drilling fluid must create a quick impermeable filter cake to seal-off all the porous formations (except the reservoir portion) as rapidly, effectively, and permanently as possible. Moreover, the drilling fluids should possess other characteristics, including: being non-damaging to the porous strata containing the hydrocarbons, being non-hazardous to the surroundings and the crew handling it, and not damaging the drilling equipment by corrosion or excessive wear^3,5–11^.

As mentioned in the above functions, one of the major additional functions of the drilling fluid is to form a stable impermeable filter cake along the interior sides of the drilled open sections of the well, especially for zones outside the production zone. A filter cake is a result of driving the liquid phase of the drilling fluid into the formation, leaving the solid particles on the side wall of the well. An ideal filter cake should be completely impermeable in order to prevent fluid losses to the formation^3,5,12^. In addition, it is recommended for the filter cake thickness to be less than or equal to 0.16 of the wellbore diameter to be removed sufficiently by one pore hole volume (PHV) of the filter cake solvent^13,14^.

However, the filter cake is considered to be one of the main cause of formation damage in the pay zone during drilling. One of the mail concern for the well completion and production engineers after drilling, is to remove the filter cake from the pay zone section to restore the productivity of the well to its original productivity^13,15,16^.

There are two main techniques for removing drilling mud filter cake. The first one relies on the mechanical action of a circulating solid-free formate brine at a high circulation rate. This mechanical action removes only about 10% of the deposited filter cake^17^. The other proposed methods use chemical reactions and include the use of live mineral and organic acids. The use of enzymes or the chelating agents are possible chemical methods for removing the filter cake^13,18,19^.

The drilling operations require continuous monitoring of the drilling fluid properties to perform multiple downhole tasks. Thus, there are two main sources of the drilling fluid solids; the added additives and the drilled formation particles. The added additives are usually designed following the American Petroleum Institute (API) procedure, in order to improve the efficiency of the drilling fluid^20–22^. On the other hand, the addition of drilled formation particles in the drilling fluid is controlled by the solid removal units consisting of shale shakers, desanders, desilters, and in some particular cases centrifuges. The use of these units is limited to reduce the cost of the drilling operations, especially if the change in the drilling fluid properties is not significant^2,3,23^.

In recent years, most of the commercial development occurred in sandstone formations. Thus, several studies were focused on evaluating the factors affecting the reservoir quality in sandstone formations before exploration, and during and after the drilling operation^14,21,24,25^. Due to the heterogeneity of sandstone reservoirs and the presence of clay minerals^21,26,27^, extensive research focus has been performed on the effect of the sand content on the drilling fluid and filter cake properties^14,28,29^. The study^28^ reported that the filter cake may form with a quartz content of up to 20% and in some portions may reach up to 40%. These filter cakes may not be susceptible to any of the abovementioned removal processes. The failure of these removal processes may occur when an accumulated quartz layer covers the weighting solids in the filter cake^25,28^.

In such cases, there is a need for a reliable process to successfully degrade quartz associated with filter cake in sandstone formations with specific steps. Based on the literature review, the objective of this work is to identify an effective solution to dissolve the complex barite filter cake (barite filter cake with high quartz content) and calcite filter cake (calcite filter cake with high quartz content) during drilling operations using water-based drilling fluids.

## Materials

The main focus was to dissolve the quartz layer in barite and calcite filter cakes. Sandstone core sample was grinded to generate the drilled cuttings that were used to simulate the quartz layer in filter cake deposition. These quartz particles were also used to evaluate the dissolution capacity of quartz during the first step of filter cake removal process.

The sandstone core sample used to generate the drilling cutting was highly rich with quartz minerals. The X-Ray Fluorescence (XRF), and X-Ray Diffraction (XRD) results show that the cuttings used in these experiments were mainly consisting of 90 wt% quartz. During the cutting separation process, the desander was used to separate the cuttings less than 45 μm in size, whereas the smaller size (up to 45 μm) was removed by the desilter^21,23^. Therefore, in this study, to ensure the same range of passed cuttings, the sandstone rock sample was crushed and then sieved using a low size 30 μm mesh to focus on the non-removable sand particles.

The composition of the barite weighted drilling fluid and calcite weighted drilling fluid is shown in Table 1. The drilling fluids formulations used in this study, prior to adding the sandstone cuttings are shown in Table 1. The sandstone cuttings were added in the drilling fluid using three different concentrations10%, 20% and 30%). The presented formulations have been used a lot as standard drilling formulations^10,13,30–32^. The density of the prepared drilling fluid was 15 ppg and 10.5 ppg for the barite and calcite weighted, respectively.

**Table 1:** Drilling fluid formulation.

Barite weighted drilling fluid			Calcite weighted drilling fluid		
Name	Unit	Description	Name	Unit	Description
Water	bbl.	0.691	Water	bbl.	0.88
Bentonite	lb.	4	Defoamer	lb.	0.08
XC-polymer	lb.	0.5	XC-polymer	lb.	1.5
BARANEX	lb.	0.25–0.50	Starch	lb.	6
KCl	lb.	20.0	KCl	lb.	80
KOH	lb.	0.5	KOH	lb.	0.3
NaCl	lb.	66	Sodium Sulfide	lb.	0.25
Barite	lb.	352.0	CaCO_3_ (50 µm)	lb.	80
CaCO_3_ (50 µm)	lb.	5.0			
Sodium sulfide	lb.	0.25–0.30			

**Figure 1:**
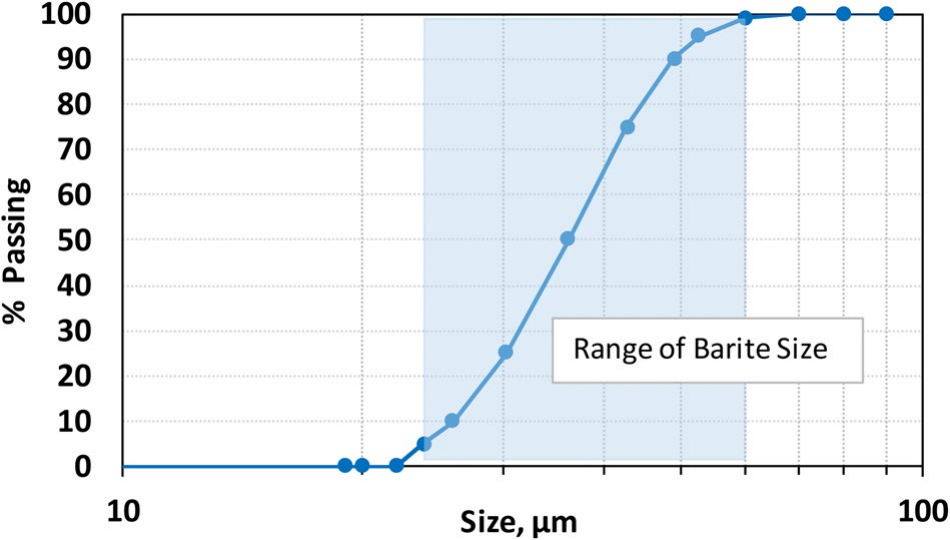
Particle size distribution of barite weighting agent.

**Figure 2:**
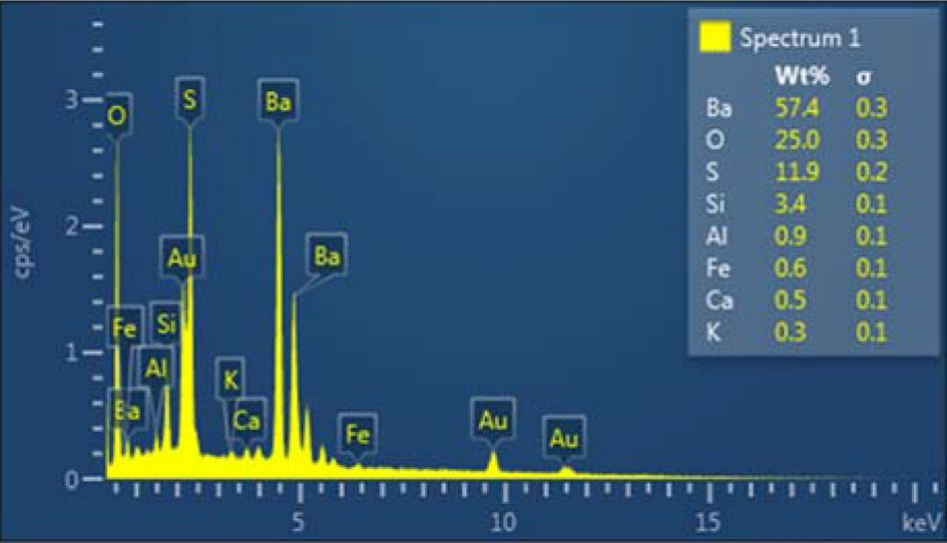
SEM–EDS of barite weighting agent.

From the removal perspective, it is extremely important to know the type of the drilling fluid weighting material of the drilling fluid. In this study, industrial grade barite (with purity > 90%), as the most common weighting agent, with particle size of 75 µm or lower was used as a weighting agent (barite base drilling fluid, Table 1). Figure 1 shows the particle size distribution analysis of the industrial grade barite used in this study, which ranged between 30–45 μm. Figure 2 shows the EDS analysis of the industrial grade barite with impurities, such as Al, Ca, Fe and Si, which are less than 3.4% or less.

## Methods

**Figure 3:**
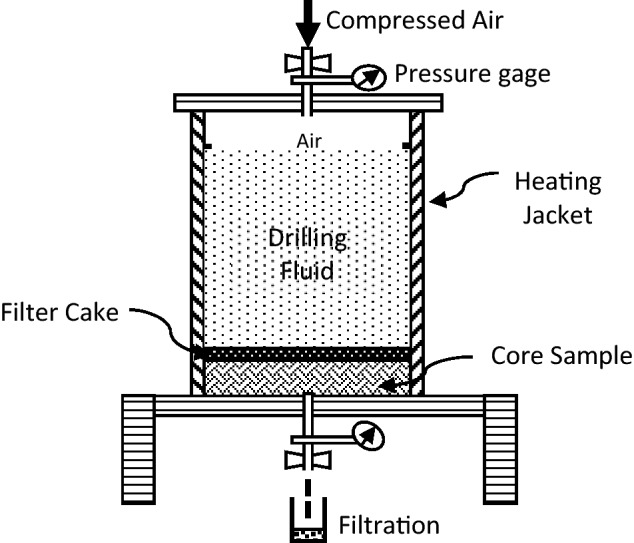
Schematic of static HPHT fluid loss system used for filter cake formation and removal experiments.

This section describes the laboratory experiments conducted to evaluate the efficiency of filter cake removal. Figure 3 shows the schematic diagram of the HPHT static fluid loss test apparatus used in this study. The filter cake was formed at the face of the ceramic disc in the filtration cell, under 500 psi differential pressure and at 200°F. Typically, the process used to evaluate the removal efficiency of the designed filter cake solvent, the filter cake is soaked in the removal solvent in the HPHT cell. However, in the current case, highly aggressive HF acid cannot be placed inside the HPHT cell due to corrosion concerns. Therefore, at the end of filter cake formation, the filter cake was carefully removed from the top of the ceramic disk. The collected solids from the filter cake were then placed in a Teflon liner cell submerged in the in-situ generated HF acid as the first stage of filter cake removal process. Hence, the purpose of the first stage is to remove the quartz particles associated with the formed filter cake. For the barite weighted base drilling fluid, the retrieved solids after HF stage (first stage) were immediately placed in the cell filled with in barite solvent (stage 2: chelating agent plus convertor agent^13,33^. The removal efficiency of the first stage was evaluated by measuring the silicate (Si) concentration using ICP-OES at the end of this stage. Overall filter cake removal efficiency was measured using the following equation:

1 $$removal \, efficiency \% = \frac{{W_{i} - W_{f} }}{{W_{i} }} \times 100$$

where Wi = initial weight of the filter cake solids, at the end of filter cake formation. Wf = final weight of the filter cake solids at the end of stage 2.

### In-situ acid generation

In-situ HF acid generation is accomplished by reacting an acid precursor, namely ammonium fluoride (NH_4_F), with a strong oxidizer, sodium bromate (NaBrO_3_) which is an endothermic reaction. The heat required for this reaction to take place is 100 °C, which is supplemented by another exothermic reaction or by downhole temperature for field operation. The thermochemical reaction is accomplished by reacting ammonium chloride with sodium nitrite. The reactions are shown in Eqs. (2) and (3):

2 $$2{\text{NH}}_{4} {\text{F}} + {\text{NaBrO}}_{3} + \Delta {\text{H}}\,\left( {{\text{heat}}} \right) \to 2{\text{HF}} + {\text{NaBr}} + 3{\text{H}}_{2} {\text{O}} + {\text{N}}_{2}$$

3 $${\text{NH}}_{4} {\text{Cl}} + {\text{NaNO}}_{2} \to {\text{NaCl}} + 2{\text{H}}_{2} {\text{O}} + {\text{N}}_{2} + \Delta {\text{H}}\,\left( {{\text{heat}}} \right)$$

4 $$\left( {{\text{NH}}_{4} } \right)_{2} {\text{SO}}_{4} + {\text{NaNO}}_{2} \to {\text{Na}}_{2} {\text{SO}}_{4} + 4{\text{H}}_{2} {\text{O}} + 2{\text{N}}_{2} + \Delta {\text{H}}\,\left( {{\text{heat}}} \right)$$

By adjusting the stoichiometry of the thermochemical reaction (Eq. 3), the generation of hydrofluoric acid can be controlled. Additionally, due to the exothermic reaction the temperature in the near wellbore region could increase up to 600°F. The solubility of barite minerals in barite dissolvers increases by increasing the temperature. Thus, the heat generated by the reaction will improve the degradation and removal of the filter cake. Additionally, the produced nitrogen gas will increase the pressure around the wellbore up to 3470 psi. This increase in pressure and evolution of nitrogen gas will also have an additional effect of creating turbulence and flow towards the top of the well and thus prevent the precipitation of reaction byproducts.

When higher pressure and temperature are needed, thermochemicals shown in Eq. (4) could be used to produce twice the amount of nitrogen resulting from the reaction in Eq. (3). Also, higher temperature could be generated from reaction in Eq. (4). For the same amount of molar volumes of chemicals, the reaction in Eq. (4) could result in almost 1.5 times higher pressure and temperature compared to that from Eq.( 3).

As previously mentioned, for the barite based drilling fluid this stage must be followed by the second removal stage by loading the chelating agent along with the converting agent^13,33^. On the other hand, for the calcite weighted drilling fluid, a second stage is required to remove the weighting agent of filter cake (calcite) by the in-situ generated HCl, as shown in Eq. (5).

5 $$2{\text{NH}}_{4} {\text{Cl}} + {\text{NaBrO}}_{3} \to 2{\text{HCl}} + {\text{NaBr}} + 3{\text{H}}_{2} {\text{O}} + {\text{N}}_{2}$$

Since Eq. (4) shares reactants NH_4_Cl and NaBrO_3_ with Eqs. (2 and 3) of the first stage, the calcite-quartz filter cake removal can be conducted in a single step by adding an excess amount of NH_4_Cl and NaBrO_3_ during the first stage to produce excess HCl acid. Also, part of these reactants generates in-situ HF to dissolve quartz in the filter cake as described in the first stage, while the excess NH_4_Cl and NaBrO_3_ generates the required amount of HCl to dissolve the calcite in the remaining filter cake. As mentioned in the first stage, the current formulation has the capability to break polymers in the filter cake due to the high pressure and high temperature generated by the thermochemical reaction.

During drilling sandstone formations, the filter cake generated during drilling operations consists of the following components:

1. Barite from the drilling fluid.
2. Quartz from the drilled formation.
3. Polymer from the drilling fluid.

Barite will be removed by injecting a slug of 20 wt% K_5_DTPA + 6 wt% K_2_CO_3_. Potassium carbonate will convert barite to barium carbonate and the DTPA chelating agent will dissolve the resulted barium carbonate. In addition, the heat generated from thermochemical reactions will enhance the efficiency of the chelating agent.

The quartz content in the filter cake during drilling sandstone formations may reach 50% of the solid content. This quartz will be removed by the in-situ generated HF acid.

The polymer coat will be removed by the temperature and pressure generated from the thermochemical reaction. In addition, the DTPA chelating agent can attack the polymer backbone and help remove the polymer.

**Figure 4:**
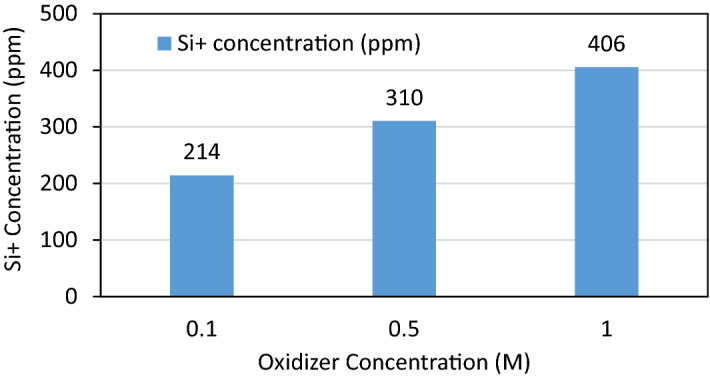
The ICP-OES results for analyzing the effluent of the reaction of the stated chemical mixtures at different oxidizer concentrations with Barite-Quartz filter cake.

Inductively Coupled Plasma (ICP-OES) analysis of the retrieved fluid sample after the first stage (quartz removal stage by HF generation flowing Eqs. (2 and 3) showed that the concentration of the silicate in the solution reached up to 400 ppm in 24 h. Moreover, repeating the same solubility test with the presence of the barite ions obtained that Si-laden HF solvent wan not affected with the presence of the barite ions. The amount of chelated silicon ions (Si^+^) depends highly on the concentration of the used oxidizer. The higher the oxidizer concentration, the higher the amount of HF generated and hence a higher amount of silica dissolved. The following chart, Fig. 4, shows different concentrations of the oxidizer that is mixed with the other stated reactants and the corresponding dissolved Si^+^ ions.

## Discussion

**Figure 5:**
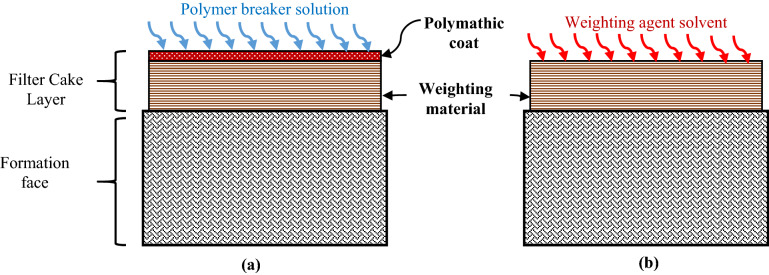
Schematic of filter cake consist of two layers (polymeric layer and weighting material). The removal process (**a**) breaks the polymer coated layer (thermochemical solution) and (**b**) dissolves the main filter cake layer (weighting material).

**Figure 6:**
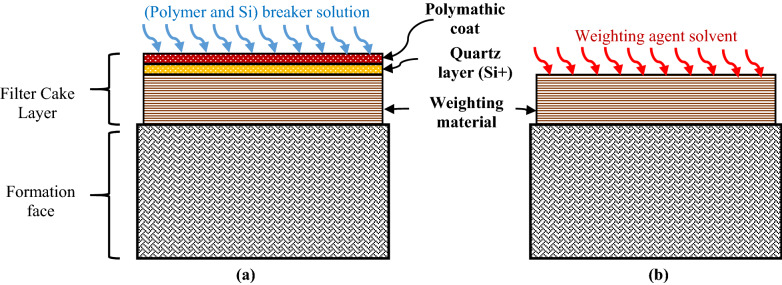
Schematic of filter cake consist of two layers (polymeric layer, fine cuttings accumulated in filter cake (quartz layer Si ^+^), and weighting material). The removal process (**a**) breaks the polymer coated layer and quartz layer (thermochemical solution) and (**b**) dissolves the main filter cake layer (weighting material).

In-situ generated HF acid can play a major role in filter cake removal process. In general, the filter cake layer is classified according to the source of the solids formed in the cake layer. It is mainly, either the drilling fluid solid additives (weighting material of the drilling fluid such as shown in Table 1) or the formation solids (cuttings) accumulated with the drilling material during the drilling operations, if they are not completely removed^21,28,29,34–36^. The filter cake solid particles are usually covered by the polymer coat and/or starch that restricts the reaction of the filter cake solids with the solvents^13,15,37^. Figures 5 and 6 show the structure of the filter cake layers. As shown in the figure, the polymer coat should be removed first in order to allow for the removal of the solids layer in the filter cake. The presence of these layers on top of weighting material will prevent its reaction with designed solvent such as chelating agent. Therefore, the polymer layer of the filter cake must be dissolved prior to the filter cake washing process^15,38^ using any of the regular polymer breakers such as α-amylase to degrade this layer^39^. Recently, Mahmoud^40^ found that using the high temperature and pressure generated using thermochemical fluid (ammonium chloride and sodium nitrate salts) was able to hydrolyze the polymer coat covering the filter cake material. According Mahmoud^40^, the exothermic reaction (Eqs. 2–4) generated temperature and pressure ensured the removal of the polymer coat around the filter cake material. Moreover, in this study, the novelty of the system to generate the heat and HF acid extends the use of this solution to dissolve the two filter cake layers (polymer layer and quartz layer) during drilling sandstone formations.

HF acid has the potential to react with and dissolve silica and silicate materials, however, the high corrosion rate of HF limits its application in the field. Therefore, the in-situ generation of HF acid will overcome this challenge and will be very beneficial in the field operations. In order to evaluate the significance of the quartz stage removal prior to dissolving the barite in the filter cake, solubility tests were conducted using different sand concentrations of 10, 20 and 30 wt%, respectively. This percentage was out of the total solid weight of 4 gm in a 100 ml of solvent in the solubility test. The solubility test was carried out under 250 °F and 500 psi using the Teflon liner cell. For barite particles, Bageri^13^ reported that the dissolution rate of barite under these conditions reached 86 wt %. The presence of the silicate in the solution (30% sand content) decreased the solubility to 60 wt%, when the same solvent was used (chelating agent ‘20% DTPA’ plus converting agent, 6%potassium carbonate) at 24 h. However this formulation (DTPA and potassium carbonate) was presented as a new formulation that has the capability to enhance the reaction with sandstone formations^41^. The reduction was due to the low rate of quartz dissolution in this formulation, which required longer soaking time to facilitate the solubility ratio. In the case of the filter cake, the presence of the sand in the structure was loaded in the top layer where the bottom layer of the filter cake was formed immediately by the weighting material (barite or calcite) before the mixing of the drilled-cutting particles with the drilling fluids. The effect of the sand layer is more significant if the designed filter cake solvent cannot penetrate this layer and has no chance to contact the weighting material of the filter cake. On the other hand, if the differential pressure in the wellbore was increased during the filter cake removal process, the removal solution will penetrate the formation after dissolving barite particles. In such case the chelated barite solvent will drop the barium in the formation and form secondary formation damage as reported by Bageri^42^. Therefore, it is desirable to dissolve the quartz layer prior to dissolving the weighting agent filter cake layer (barite or calcite).

Although, the highest removal efficiency of the barite base drilling fluid was reported to be 86%^13^ while the drilling was not contacting any quartz layer. The results as shown in Table 2 showed that using the first stage of removal (quartz removal stage) enhanced the overall removal efficiency. In the case of zero sand content using the quartz removal stage prior to barite dissolving stage produces slightly higher removal efficiency compared to the efficiency of the removal without using this stage. It is a well-known fact that barite particles have zero solubility in the HF acid. Thus, it is unlikely that the generated HF acid dissolves the low percentage solids existing in the structure of the filter cake. The source of these solids either from the drilling fluid additives used to prepare the barite base drilling fluid as shown in Table 1 (such as CaCO_3_ and KCl), or the impurities minerals associated with the barite particles as shown in SEM–EDS analysis of the barite particles that had been used as weighting agent in this drilling fluid, Fig. 2. Moreover, the generated heat during this stage (as shown Eq. 3) increased the temperature during the reaction. Thermochemical reactions presented in this study yielded additional temperature of 100 °C to the medium temperature and resulted in 1200 psi more pressure during the removal process. Hence, the dissolution rate of the barite in the chelating agent increased due to the high temperature. Temperature increase has a positive effect on the barite dissolution using chelating agent. Increasing the temperature of the medium by 100 °C due to thermochemical reaction, enhanced the barite solubility by more than 10%. Furthermore, this stage will degrade the polymer coat around the barite particle which then allow faster reaction between the weighted agent and removal solvent in the second stage. For the low sand content (10 wt.%), the overall removal efficiency using two stages removal recorded high removal efficiency due to the capability of the generated HF to dissolve the impurities in the structure of the filter cake along with this amount of Si accumulated with drilling fluid. The removal efficiency then reduced as the sand content increased. However, using two stage removal process accomplish removal efficiency reached to 80% in case of 30% sand content which still accepted compared with the removal efficiency if the first stage was ignored, as shown in Table 2.

**Table 2:** Removal efficiency of barite filter cake with and without the HF stage.

Sample ID	Sand content%	Without using quartz layer solvent	With using quartz layer solvent
		Removal efficiency %	Removal efficiency %
Sample A	0	86	95
Sample B	10	77	91
Sample C	20	68	87
Sample D	30	60	83

Interestingly, the presented solution could be easily modified for the calcite weighted drilling fluid. The observations in the calcite based drilling fluid samples differ from that in the barite system. This was expected because the weighting agent in the filter cake is soluble in the generated HF acid, therefore, it is not required to have a second stage of filter cake removal. The new formulation can react with both silicate and calcite minerals in the filter cake. In order to have a complete removal of calcite filter cake, excess amounts of NH_4_Cl and NaBrO_3_ must be added during the HF generation for producing HCl acid following the reaction in Eq. (5). It is, therefore, ensuring that HF has the ability to react with quartz in the filter cake and the generated HCl (weakens the Si reactivity acid) will dissolve the calcite particles. The result following this sequence obtained overall 100% removal efficiency for the drilling fluid consisting zero to 10% of sand content. For higher quartz concentrations, 30%, this formulation has the capability to deliver 84% overall removal efficiency.

## Conclusions

The removal efficiency of the barite filter cake using the conventional solvents is highly decreased when the sand particles intercalate with the barite layer. This occurs due to the accumulation of the sand layer with the structure of the formed filter cake and preventing the chelating agents and the catalysts from reaching the barite molecules. This arising the need for introducing a novel approach for generating HF acid that capable of dissolving the accumulated sand layer. The ammonium fluoride salt was oxidized using sodium bromate to generate in-situ HF in the presence of exothermic reaction between ammonium chloride and sodium nitrite. The exothermic reaction not only generates the heat required to accelerate the generation of HF but it also produces high pressure plus that evict any reaction precipitation.

Overall removal efficiency of the barite filter cake reached up to 85% and even more for sand content 20% and lower by using the HF stage prior to barite removal stage. The ICP results showed the capability of the acid generating system to chelate up to 400 ppm of silicon from the complex filter cake. Hence, the removal efficiency increased from 77 to 91% and from 68 to 87% and from 60 to 83% for the cases of 10, 20 and 30% sand content respectively. This enhancement in the removal efficiency is not attributed only to the HF acid generated but it is also a result of the cracks created within the filter cake by the pressure and nitrogen gas generated.

For the calcite filter cake, it is recommended to add excess amount of ammonium chloride salt in order to be oxidized and generate HCl acid. This would be of great benefit for the removal process of calcite-quartz filter cake in only one stage. The process of generating HF and HCl acids down into the well will eliminate the hazards of dealing with such corrosive acids on the surface. Moreover, the amount of the generated acids can be controlled by the amount of oxidizer injected. This reduces the problems associated with the excess of acids that may injected in other treatment jobs.
